# Supplementary figures and images for: HumGut: a comprehensive human gut prokaryotic genomes collection filtered by metagenome data
Source: Microbiome. 2021 Jul 31;9:165. doi: 10.1186/s40168-021-01114-w (PMC8325300; doi:10.1186/s40168-021-01114-w)

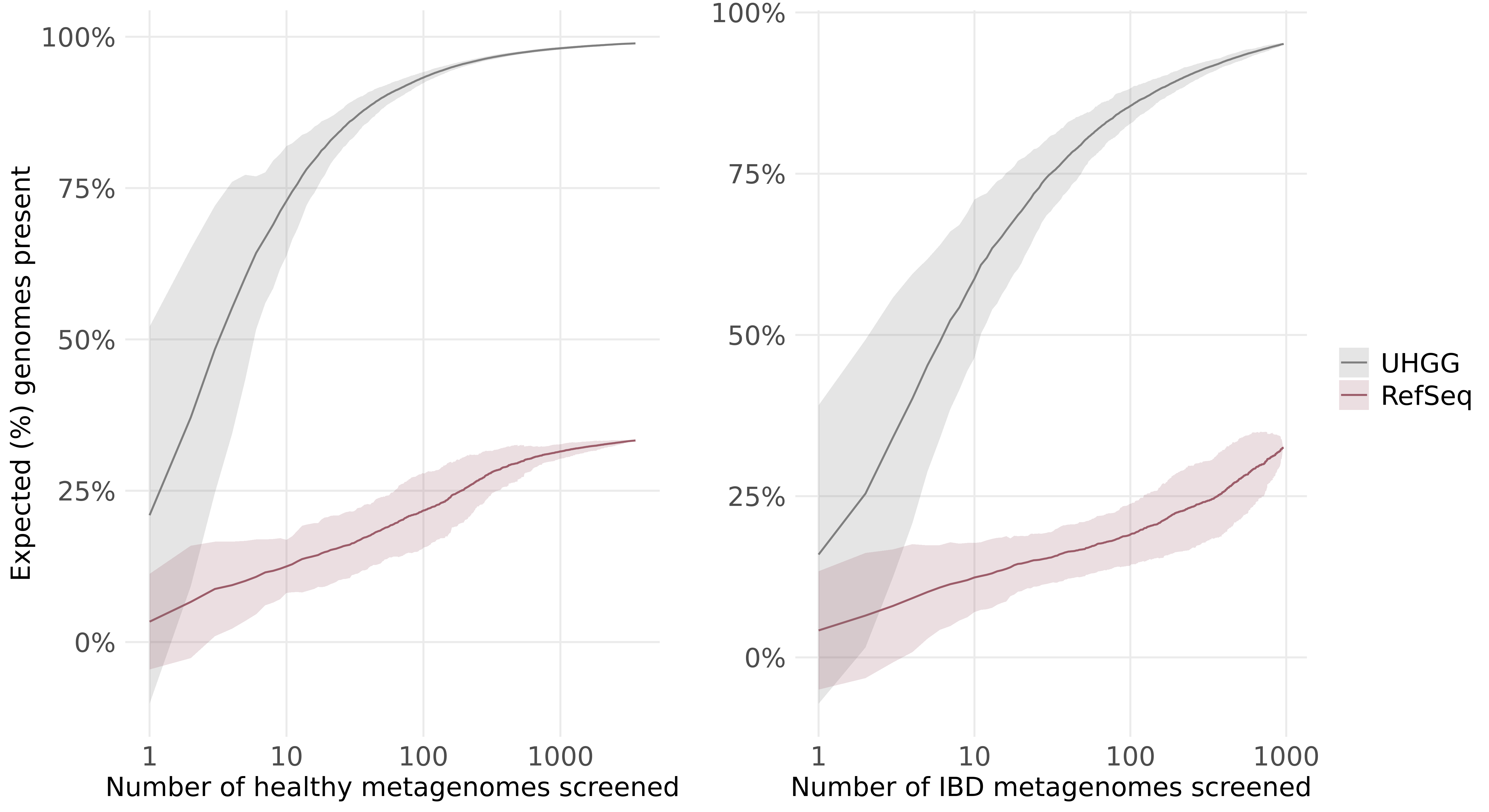

Supplement: Supplementary file 2 — Additional file 1. Figure S1. Rarefaction curves for healthy (left panel) and IBD metagenomes (right panel), showing that the number of new expected genomes flattens after screening ca. 1,000 metagenomes. [file 40168_2021_1114_MOESM2_ESM.png]

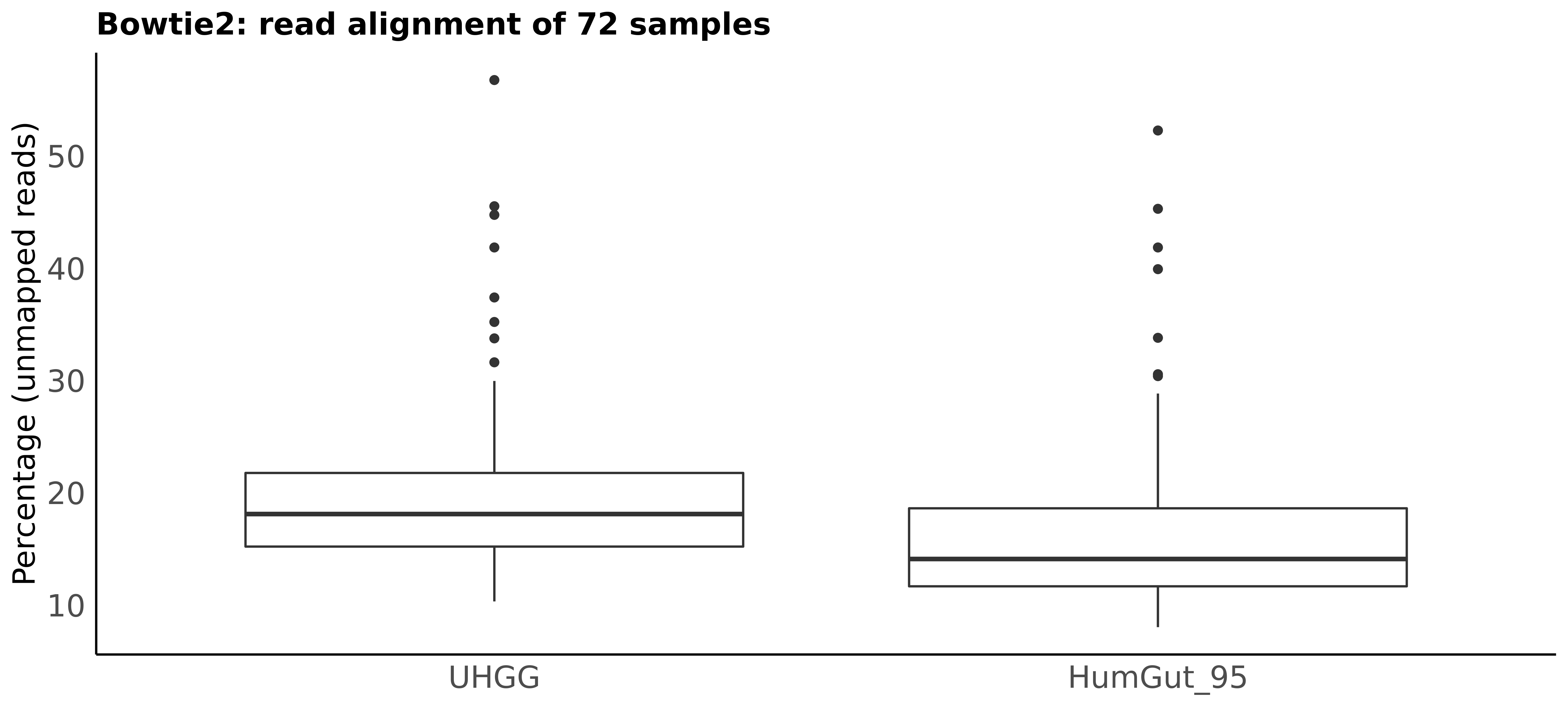

Supplement: Supplementary file 3 — Additional file 2. Figure S2. Mapping of 72 samples using Bowtie2. Y-axis shows the percentage of unmapped reads when any of the two reference index databases was used (UHGG, or HumGut_95). [file 40168_2021_1114_MOESM3_ESM.png]

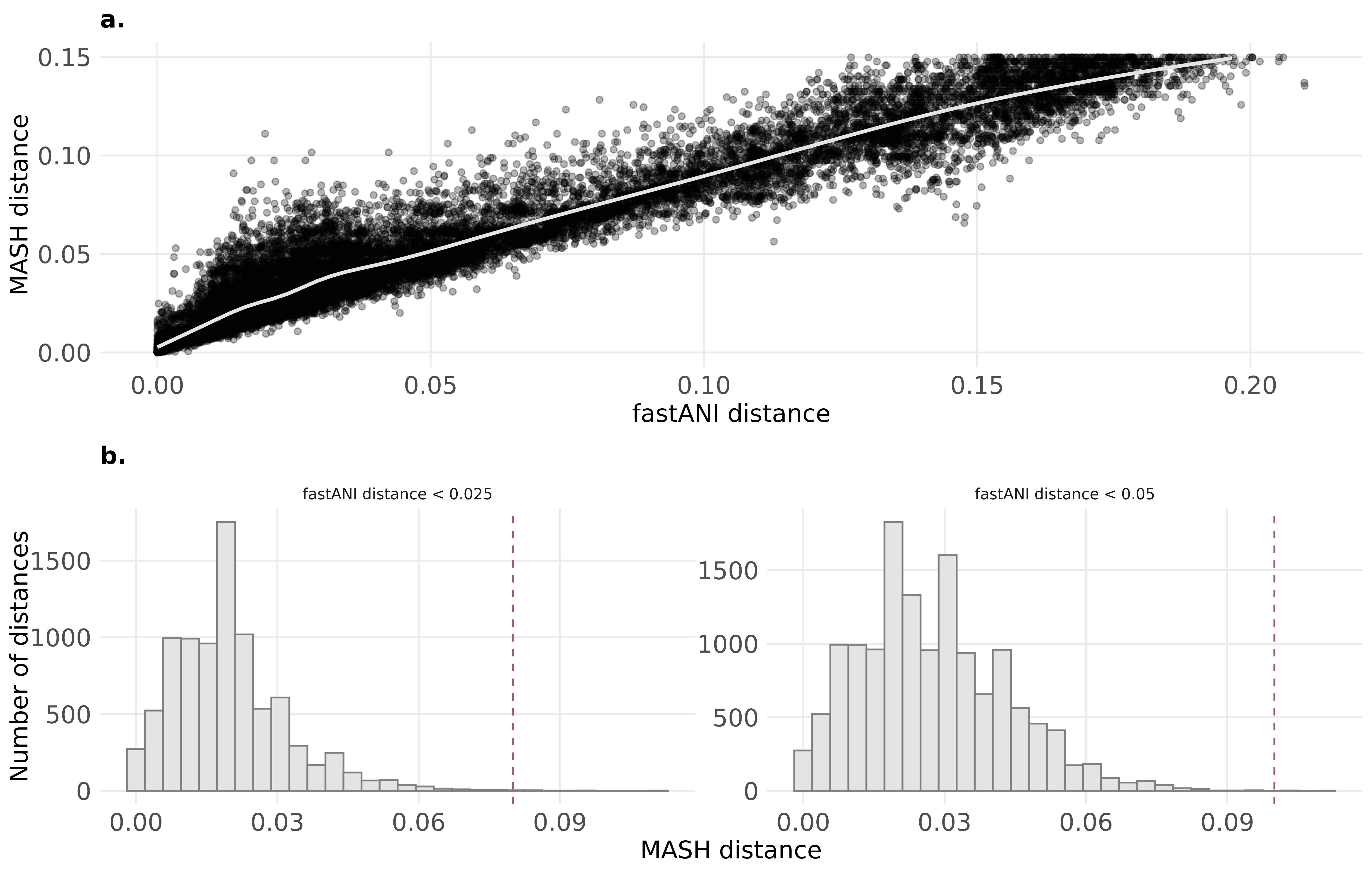

Supplement: Supplementary file 4 — Additional file 3 Figure S3. MASH and fastANI distances. a. A plot of ca. 20,000 genome distances computed with both fastANI (x-axis) and MASH (y-axis). fastANI distances tend to be a little smaller than MASH distances, they however have a substantial variance. b. The rationale behind using 0.08, and 0.1 MASH distance thresholds (vertical dashed lines) for HumGut clustering algorithm. The vast majority of fastANI distances < 0.025 have a MASH distance < 0.08 and genomes with fastANI < 0.05 have a MASH distance < 0.1. When clustering, the distance between all genomes was first computed using MASH, then only genomes with distances below the abovementioned thresholds were included to speed up fastANI computations. [file 40168_2021_1114_MOESM4_ESM.png]
